# Prevalence of Dental Fear and Its Association with Oral Health Status Among School Children in Bosnia and Herzegovina: A Cross-Sectional Study

**DOI:** 10.3390/medicina61010055

**Published:** 2025-01-01

**Authors:** Jelena Eric, Bojana Davidovic, Rasa Mladenovic, Marko Milosavljevic, Ivana Dmitruk Miljevic, Ljiljana Bjelovic, Svjetlana Jankovic, Olivera Dolic, Brankica Davidovic

**Affiliations:** 1 College of Dental Medicine, QU-Health, Qatar University, Doha P.O. Box 2713, Qatar; 2 Department of Pediatric and Preventive Dentistry with Orthodontics, Faculty of Medicine, University of East Sarajevo, Studentska bb, 73300 Foca, Bosnia and Herzegovina; bojana.davidovic@ues.rs.ba (B.D.); svjetlana.jankovic@ues.rs.ba (S.J.); 3 Department for Dentistry, Faculty of Medical Sciences, University of Kragujevac, 34000 Kragujevac, Serbia; drm.milosavljevic@yahoo.com; 4 Public Health Institution, Srpske vojske 53, 76300 Bijeljina, Bosnia and Herzegovina; drivana981@gmail.com; 5 Department of Dental Pathology, Faculty of Medicine, University of East Sarajevo, Studentska bb, 73300 Foca, Bosnia and Herzegovina; ljiljana.bjelovic@ues.rs.ba (L.B.); brankica.davidovic@ues.rs.ba (B.D.); 6 Department of Pediatric and Preventive Dentistry, Medical Faculty, University of Banja Luka, 78000 Banja Luka, Bosnia and Herzegovina; olivera.dolic@med.unibl.org

**Keywords:** children, dental fear, dental caries, oral hygiene, prevalence

## Abstract

*Background and Objective*: This study aimed to examine the prevalence of dental fear among schoolchildren in Bosnia and Herzegovina, analyze the distribution of dental anxiety by gender, age, and place of residence in relation to perceived sources of fear, and evaluate its association with oral health status. *Materials and Methods*: The sample included 355 schoolchildren between the ages of 12 and 15. Data were gathered using a self-assessment questionnaire, a brief clinical oral examination, and the Children’s Fear Survey Schedule–Dental Subscale (CFSS-DS). *Results*: Clinical examinations showed that 87.61% of the children had dental caries, with a mean DMFT score of 3.75 (SD = 2.93). The prevalence of dental caries was significantly higher in the older group compared to the younger group (*p* < 0.01). Dental fear was present in 21.7% of the children, with a mean total CFSS-DS score of 27.50 (SD = 13.85). The most feared aspect among the children was “Choking” (73.8%), followed by “Injections” (63.7%) and “The noise of the dentist drilling” (52.1%). Children with dental fear had a significantly greater number of decayed and missing teeth, higher DMFT scores, and poorer gingival health and oral hygiene compared to those without dental fear (*p* < 0.01), even after adjusting for sociodemographic factors. *Conclusions*: The study found a moderate level of dental fear among Bosnian schoolchildren, with younger children and those from urban areas showing more fear of injections. It also showed a consistent link between dental anxiety and clinical factors such as caries, gum disease, and oral hygiene, even after adjusting for sociodemographic factors.

## 1. Introduction

Dental anxiety and fear of treatment in childhood are significant public health concerns in many countries [1,2]. Early experiences in clinical settings can be challenging for children, and when these are reinforced by negative accounts from parents, caregivers, and peers about dental procedures, it can lead to a lack of trust and discomfort during their initial dental visits [3,4]. Such fear can greatly affect children’s behavior, making it difficult for them to cope with clinical situations, often resulting in irregular or delayed dental visits. This can complicate treatment and increase the risk of cavities and gum disease [1].

The percentage of children who experience dental fear or anxiety, or are at high risk for it, varies significantly across different populations, ranging from 4% to 90% [5]. For instance, a study in Hong Kong identified approximately 4% of kindergarten children as fearful based on behavioral observation during school-based outreach [6], while in Greece, 24.80% of children aged 4 to 12 were found to be anxious using a Greek version of the CFSS-DS [7]. Similarly, in Italy, the proportion of anxious children increased to 30.8% among those aged 4 to 7, using the same questionnaire [8]. This variation is influenced by factors such as the methodology employed, the criteria used, cultural differences, healthcare systems, and the age of the participants [9,10,11,12,13]. While the causes of dental fear and anxiety are complex and multifactorial, they have been linked to various socio-economic factors, parental fear, past traumatic experiences, environmental influences, and cultural beliefs [6,13,14,15,16]. Several previous studies [6,13,14] have highlighted the significant influence of gender, age, and socio-economic status on children’s levels of dental fear. These studies reported that girls and younger children generally exhibit higher levels of fear compared to boys and older children. Furthermore, children from families with lower socio-economic status and educational levels tend to experience greater dental anxiety [15].

High dental anxiety has been shown to negatively affect patients’ oral health, which in turn impacts their overall quality of life [17]. Children experiencing significant dental anxiety often demonstrate poor cooperation during dental visits, compromising treatment outcomes, and increasing stress for dental staff. This can result in more invasive procedures later, such as treatment for severe cavities, tooth extractions, surgical interventions requiring antibiotics, and even hospitalization [2,18,19]. A study of Finnish children who frequently utilized dental services revealed that 15% of them avoided seeking care due to fear of dental treatment [20]. Additionally, children with active cavities tended to be more fearful than their peers, likely due to previous negative treatment experiences [18,19].

Various methods are available for assessing dental anxiety, including physiological measures, psychometric tests, and behavioral observations [21]. One of the most commonly utilized tools is the Children’s Fear Survey Schedule–Dental Subscale (CFSS-DS) [7,8,22,23,24,25,26,27]. This questionnaire was translated into multiple languages and demonstrated good internal consistency and test–retest reliability [7,8,22,23,24,25,26,27]. Previous studies showed that children with higher CFSS-DS scores exhibited more disruptive and fearful behavior during dental treatments and were also more likely to have a history of challenging experiences [8,24,25,26].

Currently, there are limited data on the levels of dental fear among children in Bosnia and Herzegovina. Few studies have explored dental fear and anxiety among Bosnian schoolchildren [26,27], and to the best of our knowledge, none have investigated the relationship between oral health status and dental anxiety in this population. Furthermore, the prevalence of dental caries among 12- and 15-year-old schoolchildren in Bosnia and Herzegovina remains alarmingly high [28,29], especially when compared to their peers in Western European countries. These gaps in the literature highlight a crucial need for further research to understand the intersection between dental anxiety and oral health status among Bosnian children. This study aims to fill these gaps by examining the relationship between dental fear and caries prevalence, thus contributing new insights into both the psychological and clinical aspects of pediatric dental health in Bosnia and Herzegovina. Addressing these under-researched areas will not only contribute to the existing body of knowledge but also provide valuable insights for developing more effective strategies to manage dental fear and improve oral health outcomes in this population.

Therefore, this study aimed to assess the prevalence of dental fear in schoolchildren in Bosnia and Herzegovina using the CFSS-DS scale, investigate the distribution of dental anxiety by gender, age, and place of residence in relation to perceived sources of fear, and analyze the relationship between dental fear and factors such as caries, gingival health, plaque index, and malocclusion.

## 2. Materials and Methods

### 2.1. Subjects

The sample comprised 355 students from the sixth to ninth grade (approximately 12–15 years old) of both genders from the north-eastern part of Bosnia and Herzegovina. From a total of ten primary schools in the north-eastern region of Bosnia and Herzegovina, four schools were randomly selected (two urban and two rural) using an opaque envelope method. To ensure a representative sample, the schools were first categorized into two groups based on location: urban and rural. The names of the rural schools were written on separate pieces of paper and placed in one envelope, while the names of the urban schools were placed in another envelope. Two schools from each group were then randomly selected from the respective envelopes. Exclusion criteria included refusal to participate, experiencing multiple sleepless nights due to toothache, and having deciduous teeth. Verbal approval from the school principals and class teachers was obtained before the study began, and written consent was also secured from the parents. Participation was voluntary, and students had the option to withdraw from the study at any time. This research received approval from the Ethical Committee of the Faculty of Medicine in Foca, University of East Sarajevo, Bosnia and Herzegovina (01-1098/4), and was conducted in accordance with the Declaration of Helsinki.

### 2.2. Sample Size Calculation

The sample size was determined using a 95% confidence level, a 5% margin of error, and an estimated prevalence of dental fear at 26.5%, as reported by Bajric et al. (2022) [27]. The minimum required sample size was 296. Considering a response rate of 90%, at least 325 children needed to be invited to participate in the study.

### 2.3. Data Collection

Data were collected through a self-assessment questionnaire, a brief clinical oral examination, and the CFSS-DS questionnaire. The self-administered questionnaire gathered sociodemographic information, including the child’s gender (male vs. female), age (12–15), living area (urban vs. rural), father’s educational level (low, medium, high), mother’s educational level (low, medium, high), and oral health habits. Self-reported oral health habits were assessed with four questions: tooth brushing frequency (regular vs. irregular), dental flossing (no vs. yes), dental attendance (regular dental check-ups vs. symptom-oriented visits), and sugar-sweetened snack consumption (high consumer vs. low consumer).

### 2.4. Clinical Examination

All dental examinations were performed in the schools by a single trained and calibrated examiner. This examiner, with extensive experience in the field, underwent comprehensive training and calibration to ensure consistent and accurate data collection. The examination was conducted using validated clinical criteria and standardized assessment tools to reduce subjectivity and improve reliability. To assess reproducibility, a subset of 20 participants was re-examined by the same examiner after two weeks, confirming the consistency of the results. Clinical assessments occurred in the brightest classroom, using standard dental instruments such as a mouth mirror and explorer, following the criteria established by the World Health Organization (WHO). To ensure a consistent setting for the dental examinations, we conducted the assessments in the same classroom at each school. A neutral lighting setup was used for all examinations and the room was free from distractions. Prior to the examination, the children were given time to familiarize themselves with the room to help reduce any anxiety. Additionally, the examinations were scheduled at the same time each day to minimize any variability in the children’s energy levels or mood. The examiner, who was blinded to the child’s CFSS-DS score, conducted the examination. Clinical findings, along with general information, were recorded in the participants’ charts. Clinical oral examinations were employed to assess oral health status, including caries, gingival status, oral hygiene status, and type of occlusion.

Caries status was assessed using a visual–tactile examination method with standard dental instruments, including a mouth mirror and explorer, to determine the decayed, missing, filled teeth (DMFT) index. The DMFT score was calculated by summing the number of decayed, missing, and filled teeth, with participants classified as either caries-free (DMFT = 0) or having caries (DMFT ≥ 1).

The examiner assessed changes in the color and texture of the tooth surface, along with its appearance and perception to gentle probing, as well as the presence of cavitation [30]. Dark discoloration indicating cavitation, along with any roughness, chalky appearance, or softening on occlusal and smooth surfaces, was recorded. Caries on proximal surfaces were noted only if they were sufficiently advanced to create a shadow beneath the occlusal surface or had already developed cavitation.

The Löe–Silness gingival index [31] was used to assess gingival status. The gingiva around six indexed teeth was evaluated at four sites: mesiofacial papilla, facial marginal gingiva, distofacial papilla, and lingual marginal gingiva. The gingival index (GI) for each individual was calculated by summing the values for each tooth and dividing by the number of selected teeth. Scores were categorized into those with healthy gingiva (GI < 1) and those with gingivitis (GI ≥ 1). The plaque index (PI) [32] was assessed on the distofacial, facial, mesiofacial, and lingual surfaces of six indexed teeth. The PI was calculated by summing the scores for the four surfaces of each tooth and dividing the total by 4. Scores were classified into two categories: good oral hygiene (PI < 1) and poor oral hygiene (PI ≥ 1). Malocclusion was assessed according to Angle’s classification, dividing children into two groups: those with normal occlusion (Angle Class I) and those with Angle’s Class II or III malocclusions [33].

### 2.5. Survey

The level of dental fear among the children was assessed using the CFSS-DS, which has previously been validated in Bosnia and Herzegovina [25]. The CFSS-DS consisted of 15 items that addressed various aspects of dental treatment, with scores ranging from 1 (not afraid at all) to 5 (very afraid) on a Likert scale. The total score ranged from 15 to 75, with a cut-off score of 37, which also accounted for a population exhibiting latent dental fear. The cut-off score can also be justified in the context of latent dental anxiety, which refers to underlying or subclinical anxiety that may not be immediately visible but could manifest in future dental visits. Children with a CFSS-DS score of 38 or higher were classified as dentally anxious [2,13,23].

Instructions for completing the CFSS-DS were provided, and the questionnaires were completed independently by the participants. Children were instructed not to share answers or complete the questionnaire in groups.

### 2.6. Statistical Analysis

Data were collected and analyzed using the Statistical Package for Social Sciences (SPSS version 22.0, Inc., Chicago, IL, USA). Descriptive statistics, including means and standard deviations, were calculated for all variables related to dental fear, DMFT, GI, PI, and malocclusion. An independent T-test was conducted to assess differences in DMFT scores based on gender, age, and place of residence, as well as to examine dental fear score across these same demographics among the children. A chi-square test was utilized to evaluate differences in gingival and oral hygiene status by gender, age, and place of residence and the presence of dental fear across these same factors. Logistic regression models were employed to investigate both unadjusted and adjusted associations between the prevalence of dental fear (CFSS DS score ≥ 38) and clinical indicators (number of decayed, missing, and filled teeth, DMFT score, GI, PI, and malocclusion) among children. The adjusted models accounted for the effects of age, gender, and place of residence on the relationship between dental fear and clinical indicators. We also evaluated the associations between the CFSS-DS score and clinical indicators among children using non-parametric tests, as the CFSS-DS score did not follow a normal distribution. The Spearman and partial correlation coefficients were applied for the unadjusted and adjusted (controlling for age, gender, and place of residence) associations, respectively.

## 3. Results

A total of 395 children were invited to participate in the study. On the day of the clinical examination, ten children were absent, eight children declined to participate, and seven children were excluded based on the inclusion criteria. Additionally, 15 questionnaires had incomplete or invalid responses, resulting in 355 valid questionnaires and a response rate of 89.87%. The sample included 53.5% girls and 46.5% boys, with 51.3% being older children (approximately 15 years old), living in the north-eastern part of Bosnia and Herzegovina (both urban and rural areas). In terms of behavioral variables, 67.3% of children brushed their teeth irregularly, and only 31.8% used dental floss (Table 1).

Clinical oral examinations revealed that 87.61% of the children had dental caries, with a mean DMFT score of 3.75 (SD = 2.93). The prevalence of dental caries was significantly higher in the older group compared to the younger group (*p* < 0.01) (Table 2). No statistically significant differences were found in the number of decayed, missing, and filled teeth, DMFT, or Significant Caries Index (SiC) score between girls and boys (*p* > 0.05). However, a statistically significant difference was observed in the number of filled teeth between children living in urban and rural areas (*p* < 0.05). The gingival status showed no significant differences based on gender, age, or place of residence. However, older children demonstrated notably poorer oral hygiene and a higher prevalence of malocclusions compared to younger children (Table 3). Children from urban areas had a higher prevalence of gingivitis and malocclusions, although no significant difference was observed when compared to children from rural areas (*p* > 0.05).

The prevalence of dental fear was observed in 21.7% of the children, with a mean total CFSS-DS score of 27.50 (SD = 13.85), ranging from a minimum of 15 to a maximum of 75. Dental fear was more prevalent among girls, younger children, and those living in urban areas (Table 4), although the differences were not statistically significant.

The most feared variable among the children was “Choking” (73.8%), followed by “Injections” (63.7%) and “The noise of the dentist drilling” (52.1%). Girls exhibited higher levels of dental fear and anxiety than boys for the following variables: “Doctors”, “Injections”, “Having somebody examine your mouth”, “Having a stranger touch you”, “The noise of the dentist drilling”, “Having somebody put instruments in your mouth”, and “Having to go to the hospital”, showing statistical significance (Table 5). Furthermore, younger children were more fearful of injections and going to the hospital than older children, while children from urban areas were more fearful of injections compared to their peers from rural areas (*p* < 0.05).

Children with dental fear had a significantly greater number of decayed and missing teeth, as well as higher DMFT scores, compared to those without dental fear. Similar patterns were observed regarding gingival status and oral hygiene, with children experiencing dental fear showing a higher prevalence of moderate to severe gingival inflammation and poorer oral hygiene (Table 6). These significant associations were also reflected in the correlations between CFSS-DS scores and clinical measures. In each case, groups with poorer clinical outcomes were associated with higher CFSS-DS scores, indicating increased dental fear (*p* < 0.05). After controlling for age, gender, and place of residence, the number of decayed teeth showed the strongest correlation with the CFSS-DS score (r = 0.27), followed by the DMFT score. Children with higher DMFT scores were 2.63 times more likely (95% CI: 1.53–4.54) to experience dental fear compared to their peers with lower DMFT scores. Additionally, children with gingivitis and poor oral hygiene were 4.95 times (95% CI: 1.49–16.47) and 4.93 times (95% CI: 2.04–11.91) more likely, respectively, to report dental fear than their peers after adjusting for sociodemographic factors. Overall, controlling for these sociodemographic covariates did not significantly alter the estimates of the unadjusted associations; in fact, it strengthened the associations with the number of decayed and missing teeth, DMFT scores, and the GI and PI indices.

## 4. Discussion

Despite the implementation of pain management strategies in dental visits and an increasing awareness among dentists about the significance of establishing trust with patients, dental fear remains a significant challenge for both dental staff and patients [5,9,11]. This study has demonstrated that the level of dental fear experienced by adolescents in Bosnia and Herzegovina significantly influenced their oral health. Additionally, clinical indicators of oral function—such as the number of decayed, missing, and filled teeth, along with gum disease and oral hygiene—were notably linked to dental fear.

The average CFSS-DS score reported in this study was 27.50 (SD = 13.85), which aligned with other studies [7,22,23,27,34,35]. In contrast, much higher mean scores were observed among adolescents in Italy, Brazil, and the USA [8,24,36]. These differences may be due to cultural variations between countries, levels of development, access to medical and dental services, financial resources of patients, parenting practices, and the awareness of children and their parents regarding oral health. Generally, low-income and developing nations tend to have poorly organized and executed healthcare systems, resulting in children seeking care only for urgent treatments rather than for preventive measures. The healthcare system in Bosnia faces several challenges, including limited access to preventive dental care, a shortage of resources, and a lack of widespread public health initiatives aimed at educating the population on the importance of preventive measures. Consequently, many children and adolescents in Bosnia tend to seek dental care only when faced with urgent problems, such as pain or visible dental issues, rather than focusing on regular preventive visits [27]. Fear of treatment often leads to delayed or avoided preventive visits, which, combined with the high prevalence of dental caries, creates a vicious cycle of untreated oral health issues, worsening the overall oral health outcomes in this population [27].

While dental fear was more common among girls, younger children, and those living in urban areas, no statistically significant differences in fear scores were found. Some studies reported that girls had higher CFSS-DS scores [36,37], whereas others noted no significant gender differences in fear levels [23,34,35]. Conversely, certain research indicated that boys may be more fearful than girls [13]. This discrepancy could be attributed to the fact that females generally experience higher levels of fear due to lower pain thresholds and reduced pain tolerance. Additionally, cultural norms suggest that men, viewed as the stronger gender, are expected to endure more discomfort and may be less forthcoming when discussing their dental fear. A recent study in an Arabic-speaking country [20] confirmed this, suggesting that Arabic boys are generally raised to be brave and are not encouraged to express their fears, unlike girls. On the other hand, a child’s age is one of the predictors that significantly influences the presence of dental fear and anxiety in children. Most studies indicated a decrease in anxiety as children grow up [6,14]. It is assumed that dental fear in young children arises from a fear of the unknown, while cognitive abilities develop with age, leading to a greater understanding, coping, and overcoming of potential fears. Consistent to our findings, several other studies have found no differences in the severity of dental anxiety and fear between different age groups [38,39]. However, some studies have concluded that dental anxiety and fear may actually increase with age [35]. These discrepancies may be explained by the presence of potential oral health issues requiring intensive dental interventions, as well as changes in the psychological state of adolescents during significant life transitions or the ongoing impact of unpleasant past experiences [2,27]. Furthermore, cultural differences play a big role in how children experience and express dental anxiety [8,23,24,27]. Factors like family beliefs about oral health, past dental experiences, and communication styles can impact how fearful children are. In some cultures, children may hide their anxiety because of stigma or societal expectations, while in others, they may feel more comfortable showing their fear [6,8,18,19,23,24,40]. Family influences are especially important, as children often mimic their parents’ attitudes toward dental care [3,38].

In the current study, children expressed the most fear regarding “Choking”, “Injections”, and “Dentist drilling”. This aligns with findings from other studies, which also identified “Choking”, “Injections”, and “Having someone put instruments in your mouth” as the most feared aspects of dental visits [1,2,41]. These results indicated that children shared similar fears related to specific dental procedures, regardless of culture [14], suggesting that invasive procedures may be significant drivers of dental anxiety [27].

According to a WHO report, dental caries affects 60–90% of schoolchildren and a significant portion of adults, highlighting the widespread nature of this public health issue, particularly in industrialized countries [42]. In Western Europe, which is considered a low-risk region, the average DMFT score is 1.7, with 40% of 12-year-olds being caries-free [43]. In contrast, Eastern Europe, classified as a high-risk area, has a higher average DMFT of 4.1 and only 10% of 12-year-olds free of caries [43]. Some studies suggested that dental fear may be a significant predictor of dental caries and could contribute to their incidence. In our study, the mean DMFT score for the entire group was 3.75 (2.93), with only 13.2% of children being caries-free. This score is considerably higher than that observed in Western European countries but aligns more closely with the rates in the Eastern European region [43]. This study also found that 32.7% of children brushed their teeth regularly, while 31.8% reported using dental floss. The discrepancy between these habits may arise from the questionnaire not specifying whether flossing was performed regularly or only occasionally. Flossing may be perceived as a complementary activity rather than a daily routine, which could explain the higher proportion of children reporting flossing use compared to those brushing regularly. Other factors, such as diet, lack of professional dental care, or improper brushing techniques, may also contribute to the high DMFT index. In the current study, the prevalence of gingivitis, as measured by the GI, was 86.5%, which is higher than reported in other countries [44,45]. Furthermore, the average DMFT, GI, and PI values were generally greater across all characteristics in the group of children with fear (CFSS-DS ≥ 38) compared to those without fear (CFSS-DS < 38). Clinical indicators such as the number of decayed and missing teeth, along with the DMFT, GI, and PI indices, were significantly associated with dental fear. For every association, better clinical dental health correlated with lower CFSS-DS scores, even after controlling for demographic and socio-economic factors. Moreover, these associations were evident not only for the CFSS-DS score but also for the presence of dental fear (CFSS-DS score ≥ 38). The significant links between DMFT and dental fear are consistent with previous studies [13,14,45]. Conversely, some studies found no significant correlation between dental fear and DMFS-defs scores [2,6,14,35].

Additionally, several studies have reported contradictory results regarding the correlation between gingival status, oral hygiene, and dental fear [46,47,48]. Some research indicated that children with dental fear tended to have a higher prevalence of gingivitis, while other studies found no significant correlation between gingival and oral hygiene status and dental fear [46,47,48] The contradictions may arise from variations in study design, such as sample size and methodologies, as well as the subjective nature of dental fear influenced by personal and cultural factors. Additionally, other variables like socio-economic status and access to dental care may further complicate the relationship, indicating the need for more research to clarify these connections.

The implications of our findings extend beyond the research setting and are highly relevant to clinical practice and public health initiatives aimed at enhancing pediatric oral health. Healthcare professionals, including pediatric dentists and primary care providers, should prioritize the early identification and management of dental fear in children to reduce its negative impact on oral health and quality of life. By cultivating a supportive environment, both parents and schools can help alleviate children’s anxiety and encourage regular dental visits through the implementation of preventive school programs and oral health education initiatives.

While our study offers valuable insights into the relationship between dental fear and oral health status among Bosnian children, several limitations should be considered when interpreting the results. The cross-sectional design prevents us from establishing causal relationships between dental fear and oral health, underscoring the need for longitudinal studies that follow the same individuals over time to reveal changes and potential causal links. Additionally, our sample consisted of schoolchildren aged 12 to 15 from four primary schools in the north-eastern part of Bosnia and Herzegovina. As a result, our findings may not be representative of the entire child population in the country, which limits their generalizability to broader populations.

## 5. Conclusions

This study found a moderate level of dental fear among Bosnian schoolchildren. The most common fears reported by the children were “Choking,” followed by “Injections” and “The noise of the dentist’s drill.” Younger children exhibited higher levels of fear regarding injections and hospital visits compared to older children, while those from urban areas were more fearful of injections than their rural counterparts. A consistent association was observed between dental fear and clinical factors, including caries, gum disease, and oral hygiene, even after adjusting for sociodemographic variables.

Clinical implications:

- Early Intervention: Behavioral counseling, relaxation techniques, and positive reinforcement can help reduce dental fear.
- Clear Explanations: Providing visual aids and clear explanations, especially regarding injections, can alleviate anxiety.
- Age-Appropriate Interventions: Focus on building trust and familiarity with the dental environment for younger children.
- Family Involvement: Engage families in managing dental anxiety by providing resources and coping strategies.
- Outreach Programs: For high-risk groups (urban or low socio-economic populations), outreach can raise awareness, promote dental education, and encourage early intervention to prevent dental fear and oral health issues.

## Figures and Tables

**Table 1:** Sociodemographic and behavioral variables of the sample (n = 355).

Variables		n (%)
Gender	Male	165 (46.5)
	Female	190 (53.5)
Age	Younger	173 (48.7)
	Older	182 (51.3)
Area	Urban	264 (74.4)
	Rural	91 (25.6)
Father’s education	Low	2 (0.6)
	Medium	226 (63.7)
	High	127 (35.8)
Mother’s education	Low	2 (0.6)
	Medium	246 (69.3)
	High	107 (30.1)
Tooth brushing	Regular	116 (32.7)
	Irregular	239 (67.3)
Dental flossing	No	242 (68.2)
	yes	113 (31.8)
Dental attendance	Regular	101 (28.5)
	Symptom-oriented	254 (71.5)
Snack intake	High consumer	256 (72.1)
	Low consumer	99 (27.9)

Values are given as a number (percentage).

**Table 2:** Comparison of decayed (D), missing (M), and filled (F) teeth, DMFT score, and SiC by gender, age, and place of residence among the sample (n = 355).

Variables	D (SD)	M (SD)	F (SD)	DMFT (SD)	SiC (SD)
Boys	1.55 (2.24)	0.33 (0.67)	1.68 (1.77)	3.56 (2.85)	7.33 (2.81)
Girls	1.64 (2.39)	0.34 (0.75)	1.93 (2.01)	3.91 (2.98)	7.37 (2.43)
*p* value	*p* > 0.05	*p* > 0.05	*p* > 0.05	*p* > 0.05	*p* > 0.05
Younger group	1.29 (1.97)	0.22 (0.60)	1.42 (1.51)	2.93 (2.29)	7.14 (2.64)
Older group	1.88 (2.58)	0.45 (0.79)	2.19 (2.15)	4.52 (3.23)	7.41 (2.57)
*p* value	***p* < 0.05**	***p* < 0.01**	***p* < 0.001**	***p* < 0.001**	***p* > 0.05**
Urban area	1.56 (2.30)	0.30 (0.67)	1.93 (1.97)	3.80 (2.95)	7.33 (2.53)
Rural area	1.69 (2.39)	0.44 (0.81)	1.47 (1.64)	3.60 (2.85)	7.41 (2.77)
*p* value	*p* > 0.05	*p* > 0.05	*p* < 0.05	***p* > 0.05**	*p* > 0.05
Total (all)	1.59 (2.32)	0.34 (0.72)	1.81 (1.91)	3.75 (2.93)	7.35 (2.57)

Values are given as a number (percentage). *p* < 0.05 indicates statistical significance. D—decayed teeth; M—missing teeth; F—filled teeth; DMFT—decayed, missing, and filled teeth; SiC—Significant Caries Index; SD—standard deviation.

**Table 3:** Comparison of gingival index (GI), plaque index (PI), and malocclusion by gender, age, and place of residence among the sample (n = 355).

Variables/Categories (n)	Boys n (%)	Girls n (%)	Younger n (%)	Older n (%)	Urban Area n (%)	Rural Area n (%)
GI < 1	23 (6.5)	25 (7.0)	27 (7.6)	21 (5.9)	36 (10.1)	12 (3.4)
GI ≥ 1	142 (40.0)	165 (46.5)	146 (41.1)	161 (45.4)	228 (64.2)	79 (22.3)
*p* value	*p* > 0.05		*p* > 0.05		*p* > 0.05	
PI <1	43 (12.1)	42 (11.8)	50 (14.1)	35 (9.9)	63 (17.7)	22 (6.2)
PI ≥ 1	122 (34.4)	148 (41.7)	123 (34.6)	147 (41.4)	201 (56.6)	69 (19.4)
*p* value	*p* > 0.05		***p* < 0.05**		*p* > 0.05	
Malocclusion	38 (10.7)	43 (12.1)	31 (8.7)	50 (14.1)	59 (16.6)	22 (6.2)
Without malocclusion	127 (35.8)	147 (41.4)	142 (40.0)	132 (37.2)	205 (57.7)	69 (19.4)
*p* value	*p* > 0.05		***p* < 0.05**		*p* > 0.05	

Values are given as a number (percentage). *p* < 0.05 indicates statistical significance. GI—gingival index; PI—plaque index.

**Table 4:** CFSS-DS score by gender, age, and place of residence among the sample (n = 355).

CFSS-DS	Boys n (%)	Girls n (%)	Younger Group n (%)	Older Group n (%)	Urban Area n (%)	Rural Area n (%)
< 38	135 (38.0)	143 (40.3)	133 (37.5)	145 (40.8)	202 (56.9)	76 (21.4)
≥ 38	30 (8.5)	47 (13.2)	40 (11.3)	37 (10.4)	62 (17.5)	15 (4.2)
*p*	*p* > 0.05		*p* > 0.05		*p* > 0.05	

Values are given as a number (percentage). CFSS-DS—Children’s Fear Survey Schedule–Dental Subscale score; *p* > 0.05 indicates no statistical significance.

**Table 5:** Mean CFSS-DS item scores and standard deviations for all children by gender, age, and place of residence among the sample (n = 355).

Questions	Gender			Age			Place of Residence			Total
Are You Afraid of ...	Boys (165) Mean (SD)	Girls (190) Mean (SD)	*p* Value	Younger Group (173) Mean (SD)	Older Group (182) Mean (SD)	*p* Value	Urban Area (264) Mean (SD)	Rural Area (91) Mean (SD)	*p* Value	All (355) Mean (SD)
1. Dentists	1.82 (1.27)	2.09 (1.38)	*0.058*	1.94 (1.38)	1.99 (1.31)	*0.713*	1.97 (1.38)	1.97 (1.23)	*0.987*	1.97 (1.33)
2. Doctors	1.45 (1.04)	1.83 (1.31)	***0.003***	1.77 (1.35)	1.54 (1.03)	*0.078*	1.68 (1.23)	1.57 (1.09)	*0.451*	1.65 (1.20)
3. Injections	2.16 (1.47)	2.92 (1.52)	***0.000***	2.73 (1.61)	2.41 (1.49)	***0.050***	2.67 (1.58)	2.27 (1.44)	***0.038***	2.57 (1.55)
4. Having somebody examine your mouth	1.30 (0.75)	1.57 (0.94)	***0.003***	1.39 (0.71)	1.49 (0.99)	*0.297*	1.48 (0.91)	1.33 (0.73)	*0.151*	1.44 (0.86)
5. Having to open your mouth	1.24 (0.75)	1.33 (0.88)	*0.308*	1.22 (0.71)	1.35 (0.92)	*0.150*	1.30 (0.84)	1.25 (0.78)	*0.672*	1.28 (0.83)
6. Having a stranger touch you	1.41 (0.89)	1.77 (1.05)	***0.000***	1.53 (0.87)	1.68 (1.11)	*0.157*	1.63 (1.01)	1.52 (0.93)	*0.338*	1.60 (0.99)
7. Having somebody look at you	1.23 (0.67)	1.34 (0.77)	*0.153*	1.24 (0.54)	1.34 (0.88)	*0.237*	1.28 (0.71)	1.32 (0.81)	*0.668*	1.29 (0.74)
8. The dentist drilling	1.98 (1.41)	2.24 (1.43)	*0.079*	2.19 (1.51)	2.05 (1.33)	*0.351*	2.14 (1.44)	2.07 (1.35)	*0.685*	2.12 (1.43)
9. The sight of the dentist drilling	1.65 (1.20)	1.77 (1.29)	*0.370*	1.82 (1.37)	1.62 (1.12)	*0.134*	1.74 (1.28)	1.64 (1.15)	*0.507*	1.71 (1.25)
10. The noise of the dentist drilling	1.88 (1.31)	2.18 (1.36)	***0.040***	1.99 (1.27)	2.09 (1.43)	*0.463*	2.05 (1.35)	2.01 (1.33)	*0.797*	2.04 (1.34)
11. Having somebody put instruments in your mouth	1.93 (1.34)	2.26 (1.42)	***0.023***	2.21 (1.43)	2.01 (1.35)	*0.183*	2.11 (1.40)	2.11 (1.36)	*0.982*	2.11 (1.39)
12. Choking	2.64 (1.63)	2.85 (1.47)	*0.203*	2.85 (1.53)	2.66 (1.57)	*0.262*	2.75 (1.53)	2.77 (1.63)	*0.919*	2.75 (1.55)
13. Having to go to the hospital	1.56 (1.18)	1.99 (1.31)	***0.001***	2.01 (1.46)	1.59 (1.01)	***0.002***	1.84 (1.28)	1.65 (1.20)	*0.203*	1.79 (1.26)
14. People in white uniform	1.30 (0.89)	1.29 (0.82)	*0.980*	1.25 (0.78)	1.34 (0.91)	*0.371*	1.31 (0.85)	1.26 (0.87)	*0.677*	1.30 (0.85)

**Table 6:** Relationship between dental fear level and clinical status among the sample (n = 355).

Prevalence of Dental Anxiety					Overall CFSS-DS Score	
Variables	No Dental Anxiety N (SD)	With Dental Anxiety N (SD)	Crude OR (95% CI)	Adjusted OR ^†^ (95% CI)	Spearman Correlation	Partial Correlation ^†^
Decayed teeth	1.32 (2.08)	2.57 (2.85)	2.40 (1.41–4.09) **	2.63 (1.53–4.54) ***	0.199 ***	0.27 **
Missing teeth	0.29 (0.67)	0.49 (0.84)	1.91 (1.09–3.34) *	2.20 (1.23–3.96) **	0.094	0.107
Filled teeth	1.81 (1.87)	1.83 (2.06)	0.94 (0.56–1.59)	0.95 (0.56–1.62)	−0.050	0.000
DMFT	3.43 (2.83)	4.90 (2.97)	4.64 (1.40–15.37) **	4.81 (1.45–16.04) **	0.196 ***	0.248 ***
GI	0.84 (0.37)	0.96 (0.20)	4.76 (1.44–15.78) *	4.95 (1.49–16.47) **	0.070 ***	0.203 ***
PI	0.72 (0.45)	0.92 (0.27)	4.70 (1.96–11.25) ***	4.93 (2.04–11.91) ***	0.126 *	0.218 ***
Malocclusion	1.77 (0.42)	1.77 (0.43)	0.96 (0.53–1.75)	0.93 (0.51–1.70)	−0.032	−0.033

DMFT (decayed, missing, and filled teeth) score; GI—gingival index; PI—plaque index. * *p* < 0.05; ** *p* < 0.01; *** *p* < 0.001. ^†^ Adjusted for age, gender, and place of residence.

## Data Availability

The data presented in this study are available on request from the corresponding author.
